# RETRACTION: Oncological Results in Women With Wound Complications Following Mastectomy and Immediate Breast Reconstruction: A Meta‐Analysis

**DOI:** 10.1111/iwj.70554

**Published:** 2025-04-18

## Abstract

**RETRACTION:** CuiW. and XieY. , “Oncological Results in Women With Wound Complications Following Mastectomy and Immediate Breast Reconstruction: A Meta‐Analysis,” International Wound Journal 20, no. 5 (2023): 1361–1368, 10.1111/iwj.13982.36336978 PMC10088858

The above article, published online on 06 November 2022, in Wiley Online Library (http://onlinelibrary.wiley.com/), has been retracted by agreement between the journal Editor in Chief, Professor Keith Harding; and John Wiley & Sons Ltd. Following an investigation by the publisher, all parties have concluded that this article was accepted solely on the basis of a compromised peer review process. In addition, the investigation found significant textual overlap between this article and another article by different authors [1]. The editors have therefore decided to retract the article. The authors did not respond to our notice regarding the retraction.

References

[1] HanC. , YangF. , GuoS. , and ZhangJ. , "Hypertonic Saline Compared to Mannitol for the Management of Elevated Intracranial Pressure in Traumatic Brain Injury: A Meta‐Analysis,” Frontiers in Surgery 8 (2021): 10.3389/fsurg.2021.765784.PMC877698835071311

**RETRACTION:** W. Cui and Y. Xie , “Oncological Results in Women With Wound Complications Following Mastectomy and Immediate Breast Reconstruction: A Meta‐Analysis,” International Wound Journal 20, no. 5 (2023): 1361–1368, 10.1111/iwj.13982.36336978 PMC10088858

The above article, published online on 06 November 2022, in Wiley Online Library (http://onlinelibrary.wiley.com/), has been retracted by agreement between the journal Editor in Chief, Professor Keith Harding; and John Wiley & Sons Ltd. Following an investigation by the publisher, all parties have concluded that this article was accepted solely on the basis of a compromised peer review process. In addition, the investigation found significant textual overlap between this article and another article by different authors [1]. The editors have therefore decided to retract the article. The authors did not respond to our notice regarding the retraction.

**References**

[1] C. Han , F. Yang , S. Guo , and J. Zhang , "Hypertonic Saline Compared to Mannitol for the Management of Elevated Intracranial Pressure in Traumatic Brain Injury: A Meta‐Analysis,” Frontiers in Surgery 8 (2021): 10.3389/fsurg.2021.765784.PMC877698835071311

